# Pulmonary cryptococcosis with headache as the first presentation: A case report

**DOI:** 10.1515/biol-2025-1119

**Published:** 2025-08-12

**Authors:** Ting Xu, Shuai Luo

**Affiliations:** Department of Pathology, Zhejiang Provincial People’s Hospital Bijie Hospital, Bijie, Guizhou, P.R. China; Department of Pathology, Affiliated Hospital of Zunyi Medical University, Zunyi, Guizhou, P.R. China

**Keywords:** initial symptom, cytology, *Cryptococcus*, diagnosis, differential diagnosis

## Abstract

Pulmonary cryptococcosis (PC) is an invasive fungal infection caused by *Cryptococcus neoformans* or *Cryptococcus gattii*. Its clinical presentation and radiological findings are often non-specific, making early diagnosis challenging. Herein, the case of a 44-year-old male who presented with dizziness and headache is reported. Initial cranial magnetic resonance imaging and chest computed tomography (plain and contrast-enhanced) suggested lung cancer with brain metastasis. A definitive diagnosis was established only after a lung mass biopsy, followed by a cytological smear and histopathological analysis, confirmed PC. The patient was treated with antifungal therapy postoperatively and responded well. This case underscores the importance of considering PC in differential diagnoses to enable prompt diagnosis and treatment, potentially reducing associated mortality.

## 1 Background

Pulmonary cryptococcosis (PC) is an invasive fungal disease caused by *Cryptococcus neoformans* or *Cryptococcus gattii*. Its clinical manifestations and imaging features are often non-specific, making diagnosis challenging. PC is frequently misdiagnosed as lung cancer or tuberculosis. Delayed treatment might result in cryptococcal dissemination to the central nervous system, leading to cryptococcal meningitis, which carries a high mortality rate [1]. Thus, early diagnosis and timely intervention are crucial. However, achieving an early diagnosis remains challenging.

Herein, a case of disseminated PC presenting initially with headache is reported. *Cryptococcus* was identified through a cytological smear and histopathological biopsy, leading to a confirmed diagnosis. This case highlights the importance of raising clinical awareness to support early diagnosis and prompt treatment.

## 2 Case demonstration

A 44-year-old Han Chinese male was admitted with a 20-day history of dizziness and headache. The symptoms began without an obvious trigger and were paroxysmal, described primarily as mild pain. During severe episodes, the patient experienced a stretching sensation across the entire head, accompanied by blurred vision and unsteady posture. These episodes lasted a few minutes and resolved spontaneously, without syncope or vertigo. There was no associated visual rotation, nausea, vomiting, changes in smell or taste, limb convulsions, or faecal incontinence.

**Figure 1:**
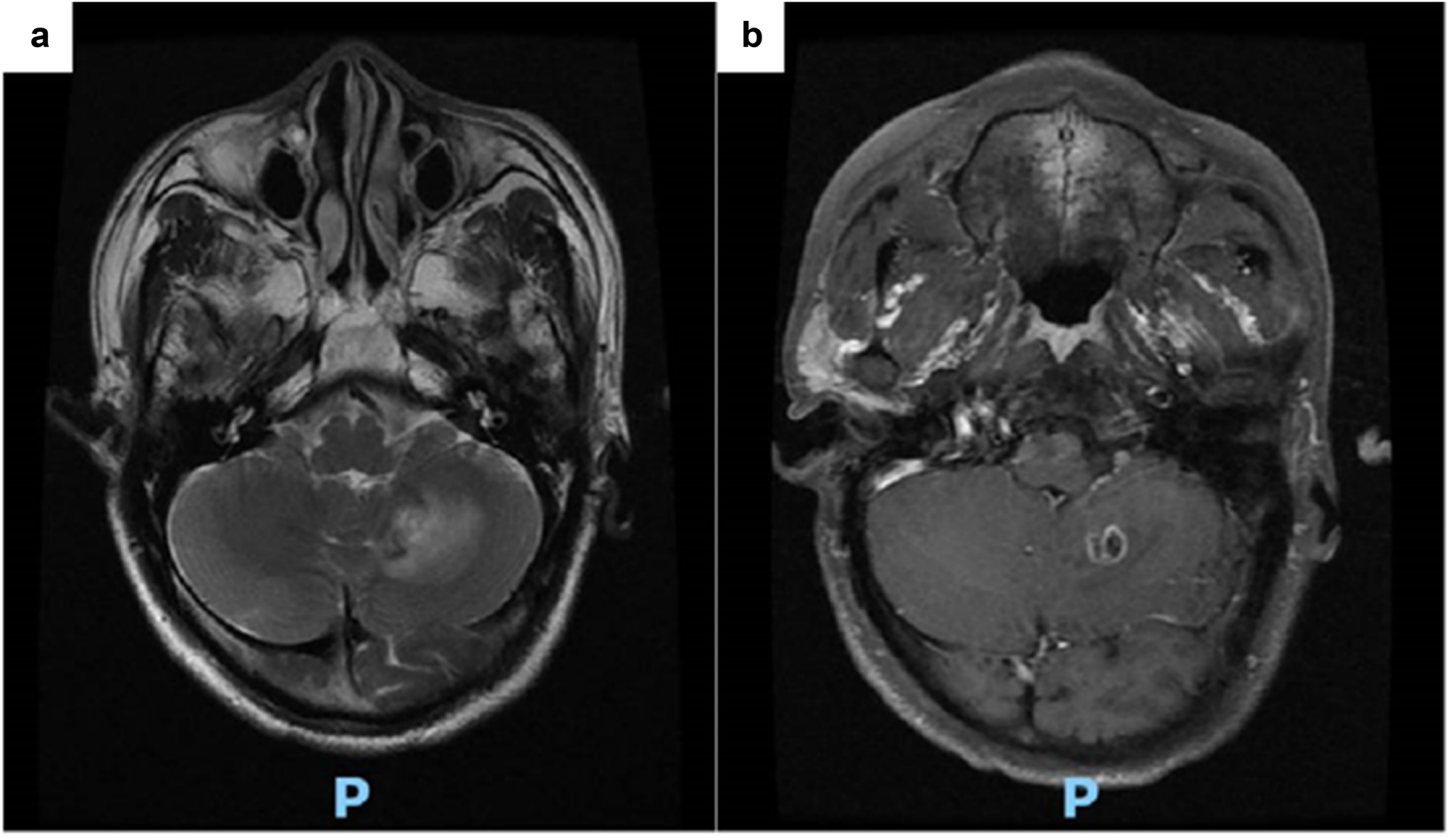
Head MRI showing irregular cystic nodules in the left cerebellum with ring enhancement on enhanced scan, and spectral spectroscopy (MRS) suggested that the lesions were mainly necrotic: (a) Enhancement and (b) MRS.

Cranial magnetic resonance imaging (MRI) (Figure 1a and b) revealed irregular cystic nodules with long T2 and T1 signals in the left cerebellum. The cyst wall was isointense, measuring approximately 16 mm × 11 mm × 13 mm. Diffusion-weighted imaging showed isointensity, while contrast-enhanced scans demonstrated ring enhancement. Additionally, a small enhancing nodule (∼3.5 mm) was observed in the interpeduncular cistern. Magnetic resonance spectroscopy (MRS) suggested predominant necrosis, raising suspicion for metastatic tumour or glioma. The patient was thus admitted with a preliminary diagnosis of a “left cerebellar tumour.” At admission, he reported generally normal mood, appetite, and sleep, though he had long-standing constipation, nocturia, and no significant weight change. Physical examination revealed a blood pressure of 144 mmHg, clear consciousness, slightly diminished direct and indirect pupillary light reflexes, blurred vision, and no other obvious abnormalities.

**Figure 2:**
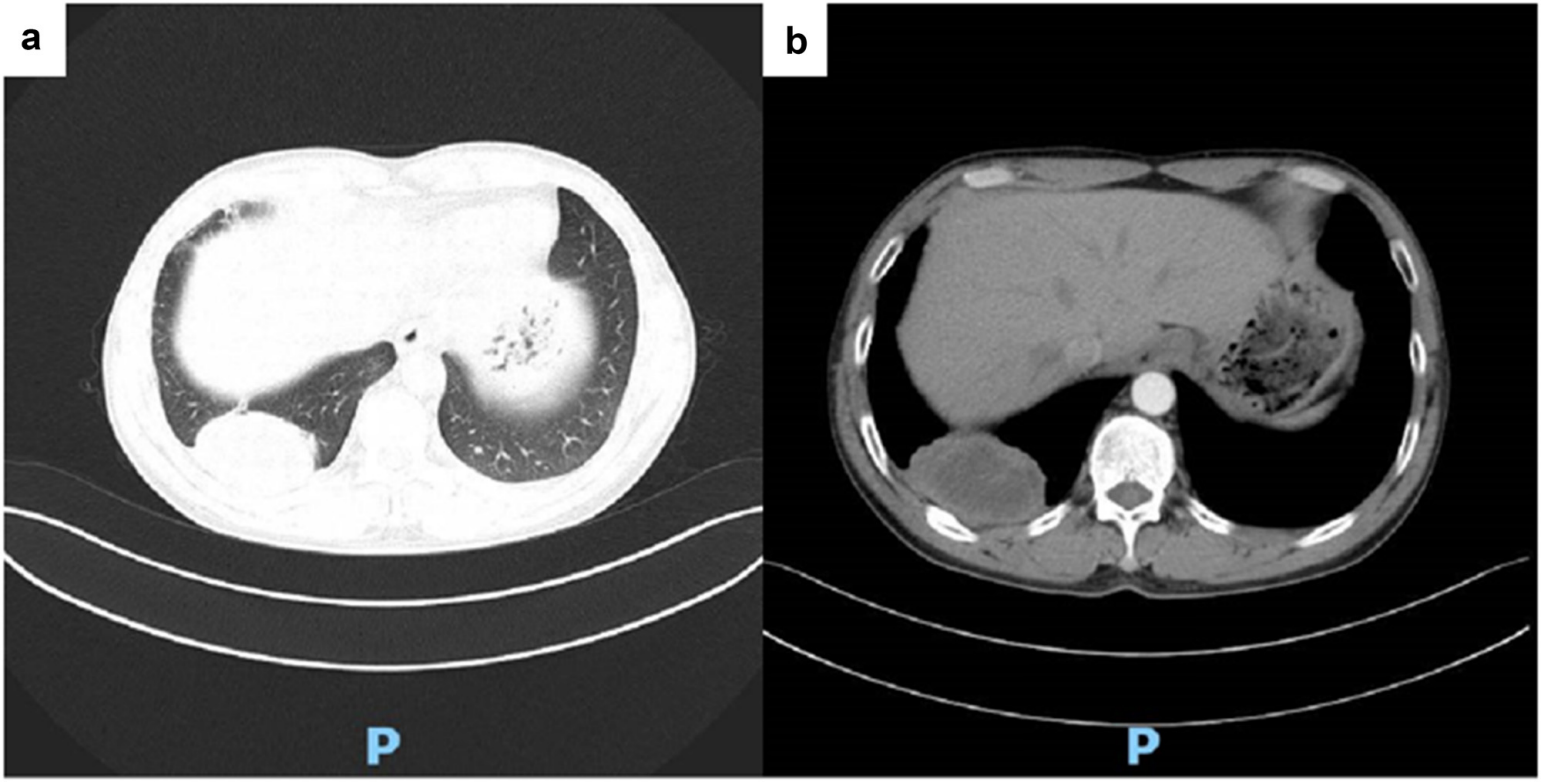
Chest CT: A mass shadow is found in the lower lobe of the right lung, with clear boundaries and uneven density, and patchy low-density shadow is found within it. (a) Plain scan and (b) enhanced arterial phase.

Given the possibility of a metastatic tumour based on cranial MRI findings, a contrast-enhanced chest computed tomography (CT) (Figure 2a and b) was performed. It revealed a 43 mm × 40 mm mass in the lower lobe of the right lung, with well-defined margins, heterogeneous density, and patchy areas of low attenuation. Mediastinal lymphadenopathy was present, with some lymph nodes appearing enlarged and calcified. The mass exhibited heterogeneous enhancement along with pleural thickening and adhesion. These imaging features were suggestive of peripheral pulmonary carcinoma.

Integrating the findings from the cranial MRI and chest CT (plain and contrast-enhanced), the initial clinical impression was lung cancer with brain metastasis. A CT-guided needle biopsy was subsequently performed to determine the pathological nature of the lesion, and specimens were submitted for conventional cytological smear and histopathological examination.

**Figure 3:**
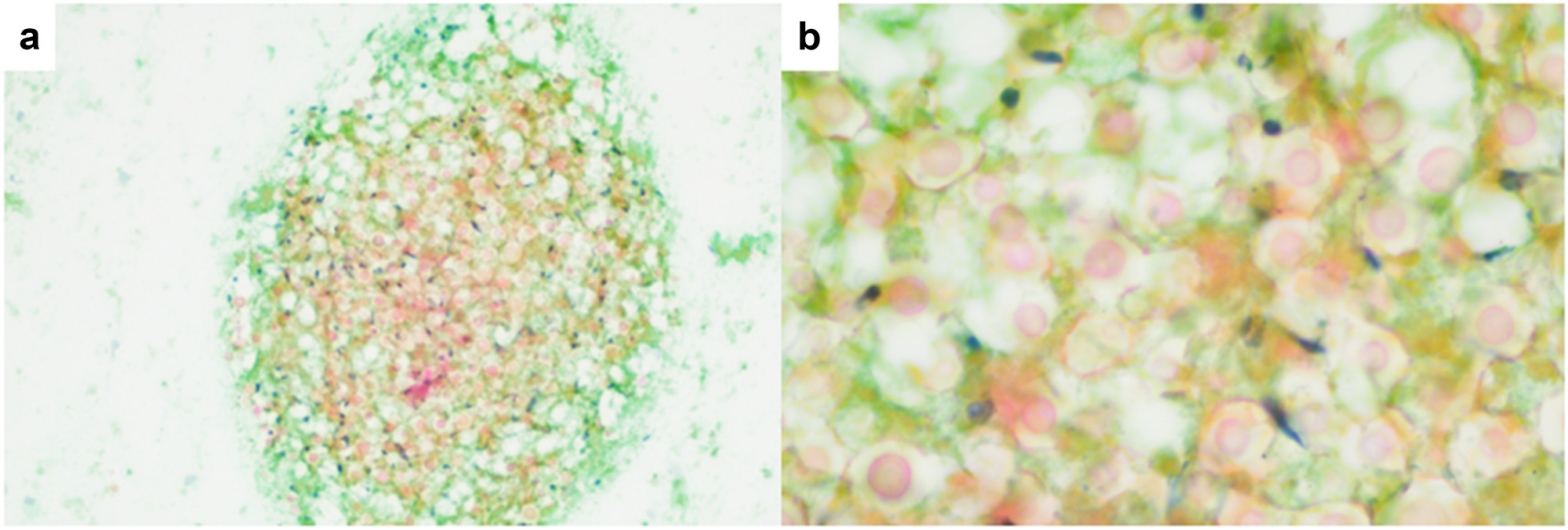
Spheroidal thellites of varying sizes were observed in traditional cytological smears, The cells were purplish red and without nuclei. Cytological Pap staining: (a) ×100 and (b) ×400.

Cytological smears (Figure 3a and b) revealed spherical microorganisms of varying sizes, (5–20 μm), distributed at regular intervals. The organisms were purplish-red, lacked nuclei, and were surrounded by thick, intensely stained walls. Prominent unstained halos were observed around each cell.

**Figure 4:**
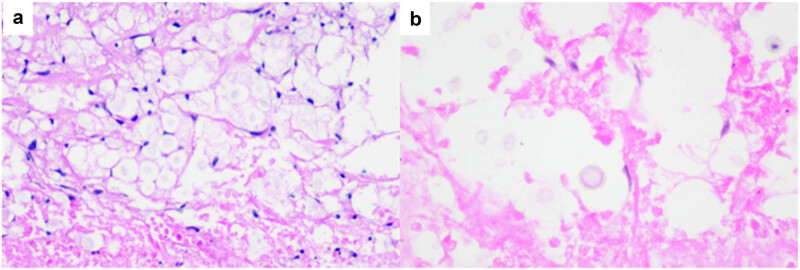
Under the microscope of histopathological biopsy, Cryptococcus are seen in the foamy cells and extracellular stroma, which are round or oval, slightly blue-stained or vacuolated, varying in size and surrounded by a transparent and refractive capsule. H&E (a) ×200 and (b) ×400.

**Figure 5:**
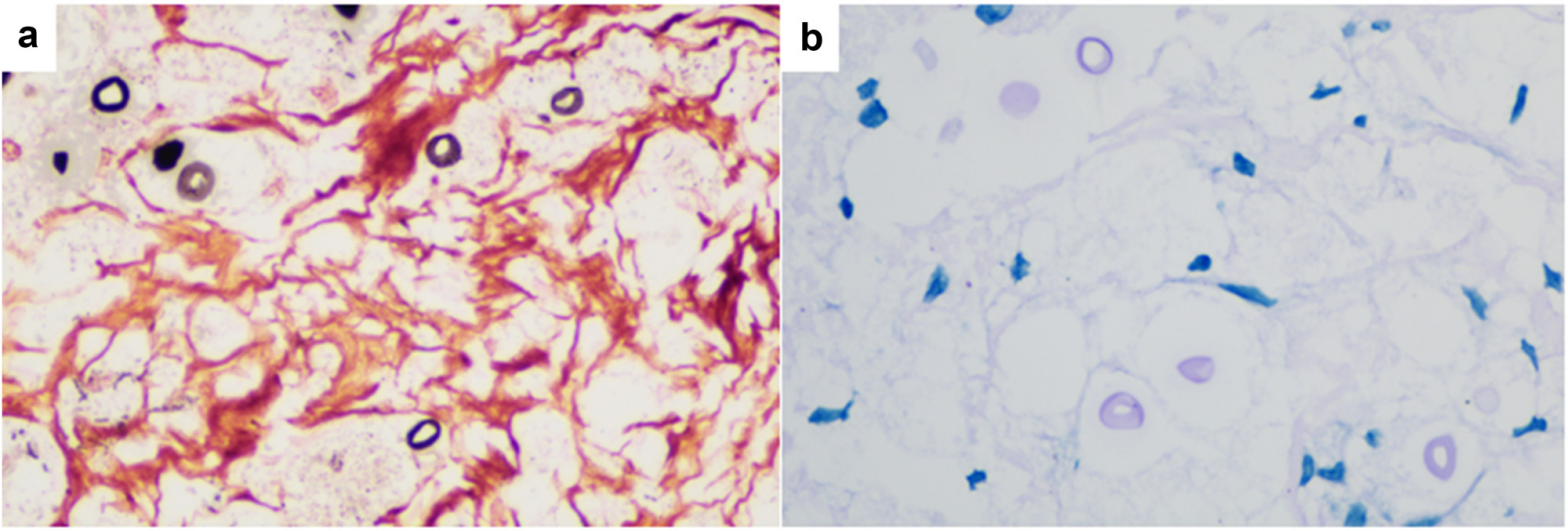
Cryptococcus can stain the fungal walls in GMS (a) and PAS (b) as purplish red and brown-black, respectively. ×400.

Histopathological examination (Figure 4a and b) revealed granuloma formation within a background of chronic inflammation, characterised by fibrous tissue hyperplasia and mild lymphocytic infiltration. Numerous *Cryptococcus* spores were observed with foamy macrophages, extracellular matrix, and alveolar spaces. The spores appeared round or oval, exhibiting bluish or vacuolated cytoplasm. Special staining with Grocott’s methenamine silver (GMS) (Figure 5a) and periodic acid-Schiff (PAS) (Figure 5b) enhanced the visualisation of the fungal cell walls, staining them brown-black and purplish-red, respectively. The spores measured 5–20 μm in diameter and were surrounded by transparent, refractive capsules, which were delineated by these stains and aided in the identification of *Cryptococcus*.

Based on the clinical presentation, cytological findings, and histopathological evidence, the patient was diagnosed with PC combined with cryptococcal meningitis.

Following the diagnosis of PC, further evaluation was conducted to exclude the presence of additional lesions. Positron emission tomography-CT revealed increased metabolic activity in the left cerebellar hemisphere, heterogeneous metabolic uptake in the right lower lobe of the lung, and elevated metabolism in the right maxillary sinus and nasopharynx. These findings were suggestive of infectious lesions.

**Figure 6:**
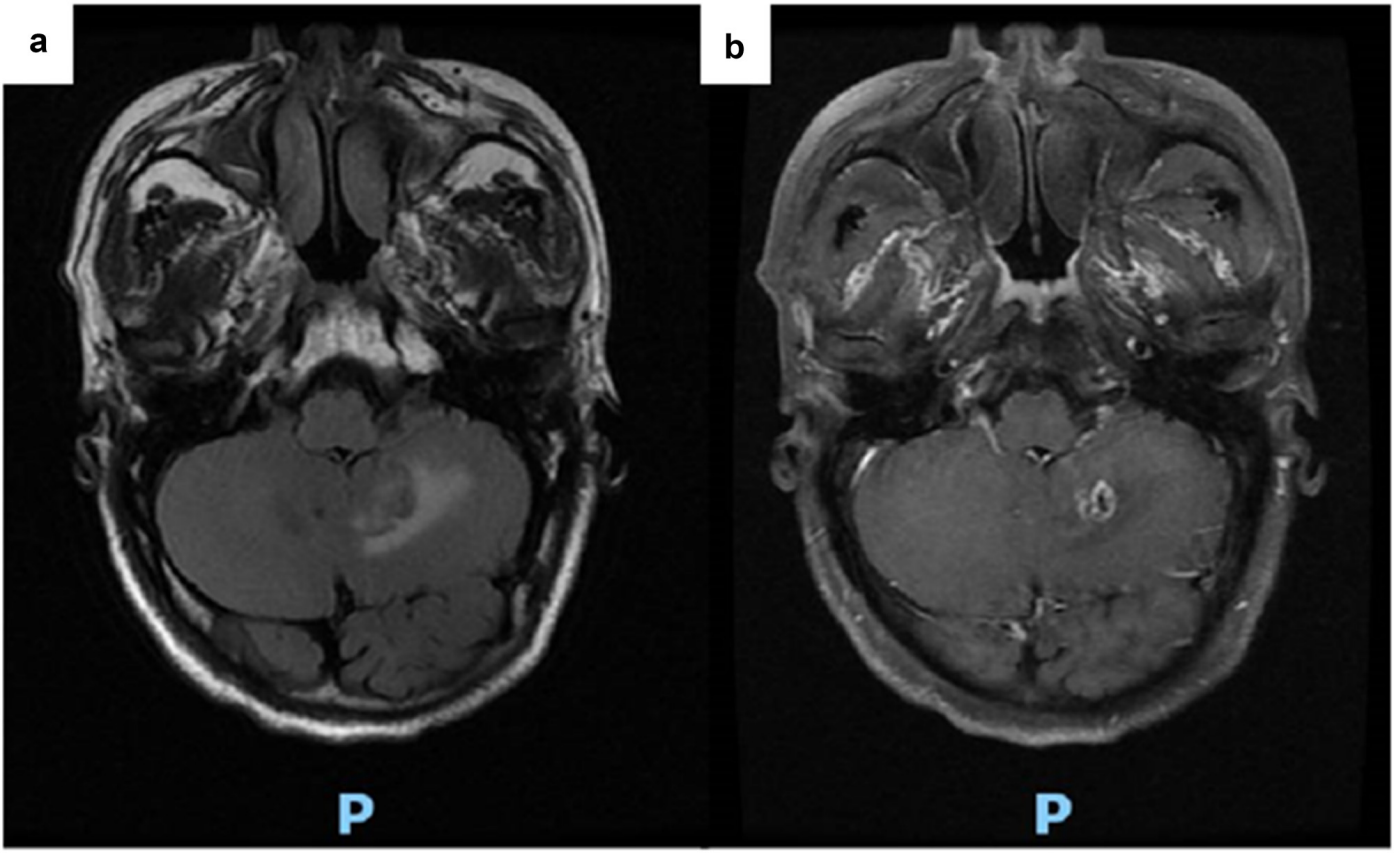
Head MRI after 1 week of antifungal therapy: Irregular ring-enhanced nodules in the left cerebellar hemisphere with surrounding patchy cerebral oedema. The lesions in the left cerebellar hemisphere are smaller than before, and the enhancement ring is thicker and more obvious than before. (a) Enhancement and (b) MRS.

The patient was initiated on antifungal therapy with amphotericin B for 1 week, improving headache and dizziness. Follow-up cranial MRI (Figure 6a and b) demonstrated an irregular ring-enhancing nodule measuring approximately 13 mm × 9 mm in the left cerebellar hemisphere, with surrounding patchy cerebral oedema. MRS showed decreased choline, creatine, and N-acetylaspartate peaks in the lesion area. Compared to previous imaging, the left cerebellar lesion had been reduced in size, while the enhancement ring appeared thicker and more defined.

After discharge, the patient continued treatment with amphotericin B and oral pentafluorouracil as adjuvant therapy. During the 9-month follow-up, the patient’s general condition remained stable, with no signs of recurrence.


**Informed consent:** Informed consent has been obtained from all individuals included in this study.
**Ethical approval:** The research related to human use has been complied with all the relevant national regulations, institutional policies, and in accordance with the tenets of the Helsinki Declaration, and has been approved by the Ethics Committee of the Affiliated Hospital of Zunyi Medical University.

## 3 Discussion

PC is a significant opportunistic infection predominantly caused by *Cryptococcus neoformans*, associated with high mortality and morbidity [2]. Radiological findings, particularly those presenting as nodules or mass-like opacities, are often indistinguishable from lung cancer in imaging studies [3]. Histopathologically, the typical manifestation involves granulomatous inflammation, which necessitates differential diagnosis from tuberculosis and sarcoidosis [4]. Therefore, PC is frequently misdiagnosed clinically and pathologically.

The clinical presentation of PC patients lacks specificity, and disease severity is closely related to the immune status of the host. Immunocompromised individuals are at a greater risk of extrapulmonary dissemination. In cases involving the central nervous system, cryptococcal meningitis might develop, often associated with a poor prognosis and high mortality [1]. In immunocompetent patients, common clinical manifestations include fever, cough, sputum production, chest pain, dyspnoea, and haemoptysis [1,5]. Notably, approximately 30% of patients might remain asymptomatic, with pulmonary lesions discovered incidentally during imaging performed for routine health examinations or unrelated medical conditions [6].

In the present case, the patient was admitted to the hospital with complaints of headache. Based on initial cranial MRI and chest CT findings, a diagnosis of lung cancer with brain metastasis was suspected. However, pathological biopsy confirmed PC. This case underscores the non-specific clinical presentation of PC, particularly when central nervous system involvement occurs, which significantly worsens patient prognosis.

Early diagnosis and timely treatment are essential for improving patient prognosis. Current diagnostic approaches include ink staining, cryptococcal culture, cryptococcal capsular antigen (CrAg) testing, and histopathological examination. Ink staining has limited sensitivity, especially in non-human immunodeficiency virus (HIV)-infected individuals, with positive detection rates of approximately 75% in HIV-infected individuals and 50% in non-HIV-infected individuals [7]. Cryptococcal culture remains the diagnostic gold standard for cryptococcal meningitis and is useful for prognostic assessment and monitoring treatment response. However, its prolonged turnaround time limits its utility for early diagnosis [8]. The lateral flow assay for CrAg detection has emerged as a rapid, cost-effective method capable of quantitative and qualitative analysis in complex samples. It is widely adopted as the primary diagnostic tool for CrAg detection worldwide. However, its inability to monitor therapeutic response based on antigen titre remains a limitation [9].

The article primarily focuses on the diagnostic value of conventional cytological smears and histopathological biopsies in detecting PC. In conventional cytological smears, *Cryptococcus* typically appears as isolated or small clusters of poorly stained, pale pink, round organisms measuring approximately 7–18 μm in diameter. These organisms might be located extracellularly or within multinucleated giant cells [10]. However, cytological diagnosis of *Cryptococcus* remains relatively uncommon due to its non-specific features.

In histopathological biopsies, cryptococcal spores are observed within foamy macrophages or alveolar spaces. These spores are usually round to oval, bluish or vacuolated, and range from 5 to 20 μm in diameter. They are characteristically surrounded by a transparent, refractive capsule. Special fungal stains such as GMS and PAS are used to enhance visualisation, staining the fungal wall dark brown-black and purplish-red, respectively, thereby highlighting the capsule more distinctly [11].

Histologically, *Cryptococcus* must be differentiated from other fungal infections, such as histoplasmosis, coccidioidomycosis, and sarcoidosis.

(1) Histoplasmosis: The organisms are intracellular, round or oval, and considerably smaller (1–5 μm; average ∼3 μm) than *Cryptococcus* [12]. They are often surrounded by a clear halo (pseudopod membrane) outside the cell, which was proven to be non-capsular based on PAS staining.
(2)Coccidioidomycosis: The spores are significantly larger (20–150 μm) than Cryptococcus spores and lack budding as a mode of reproduction.
(3) Sarcoidosis: Characteristic Schaumann bodies – concentrically laminated, round or oval structures – can be observed within multinucleated giant cells. These bodies stain dark blue and appear as black structures when stained with PAS and hexamine silver [13].

Amphotericin B remains the cornerstone of antifungal therapy for PC; however, a thorough assessment of the patient’s immune status is essential. Treatment strategies should be guided by immune competence, central nervous system involvement, disseminated cryptococcosis, and overall disease severity [14].

## 4 Conclusion

The clinical manifestations and imaging features of PC often lack specificity, and the prognosis is particularly poor in cases complicated by cryptococcal meningitis. These factors continue to pose significant challenges for early diagnosis and treatment. Cytological examination – whether via conventional smear or liquid-based cytology – offers a non-invasive and rapid alternative to histopathological analysis. Clinicians should maintain a high index of suspicion for infections such as cryptococcosis, especially when patients present primarily with headache and inconspicuous pulmonary symptoms. It is crucial to avoid premature conclusions of malignancy based solely on imaging findings. This case highlights a presentation of disseminated PC with headache as the initial symptom. The patient demonstrated favourable outcomes following prompt antifungal therapy, underscoring the importance of early diagnosis and timely intervention to reduce mortality.
